# Identification of Mechanical Properties for Titanium Alloy Ti-6Al-4V Produced Using LENS Technology

**DOI:** 10.3390/ma12060886

**Published:** 2019-03-16

**Authors:** Aleksandra Szafrańska, Anna Antolak-Dudka, Paweł Baranowski, Paweł Bogusz, Dariusz Zasada, Jerzy Małachowski, Tomasz Czujko

**Affiliations:** 1 Department of Mechanics and Applied Computer Science, Military University of Technology, Gen. W. Urbanowicza 2 St., 00-908 Warsaw, Poland; aleksandra.szafranska@wat.edu.pl (A.S.); pawel.baranowski@wat.edu.pl (P.B.); pawel.bogusz@wat.edu.pl (P.B.); 2 Department of Advanced Materials and Technologies; Military University of Technology, Gen. W. Urbanowicza 2 St., 00-908 Warsaw, Poland; anna.dudka@wat.edu.pl (A.A.-D.); dariusz.zasada@wat.edu.pl (D.Z.); tomasz.czujko@wat.edu.pl (T.C.)

**Keywords:** additive manufacturing, Ti-6Al-4V, LENS, mechanical characterization

## Abstract

This paper presents a characterization study of specimens manufactured from Ti-6Al-4V powder with the use of laser engineered net shaping technology (LENS). Two different orientations of the specimens were considered to analyze the loading direction influence on the material mechanical properties. Moreover, two sets of specimens, as-built (without heat treatment) and after heat treatment, were used. An optical measurement system was also adopted for determining deformation of the specimen, areas of minimum and the maximum principal strain, and an effective plastic strain value at failure. The loading direction dependence on the material properties was observed with a significant influence of the orientation on the stress and strain level. Microstructure characterization was examined with the use of optical and scanning electron microscopes (SEM); in addition, the electron backscatter diffraction (EBSD) was also used. The fracture mechanism was discussed based on the fractography analysis. The presented comprehensive methodology proved to be effective and it could be implemented for different materials in additive technologies. The material data was used to obtain parameters for the selected constitutive model to simulate the energy absorbing structures manufactured with LENS technology. Therefore, a brief discussion related to numerical modelling of the LENS Ti-6Al-4V alloy was also included in the paper. The numerical modelling confirmed the correctness of the acquired material data resulting in a reasonable reproduction of the material behavior during the cellular structure deformation process.

## 1. Introduction

Additive manufacturing (AM) is currently the fastest growing manufacturing method. It allows for the obtainment of products with a complex geometry and a high dimensional accuracy, as well as with high strength parameters [1]. The applied technology depends mainly on the batch material used. Products made of polymeric materials are obtained with such techniques as fused deposition modeling (FDM), where the material is a thermoplast in a circular wire; stereolitography (SLA), where the material is a light-cured resin; and selective laser sintering (SLS), where the batch material is a powdered polymer [2,3]. For metal alloys, techniques including powder bed fusion (PBF), selective laser melting (SLM), electron beam melting (EBM) and direct energy deposition (DED), for instance, laser engineered net shaping (LENS) [4,5,6,7] are used.

The tested specimens were prepared from the powder of Ti-6Al-4V alloy using LENS technology, where the metal powder is melted with a high-power laser beam, layer by layer. The technology enables repairing the damaged parts and manufacturing new fully functional elements, with properties generally comparable to those made by conventional methods [8,9,10]. As a result of high cooling rates during the process, the products have a fine-grained structure, which increases their mechanical properties. The disadvantage of these products are high surface roughness and reduced plasticity associated to the residual stress generated during the rapid material solidification. Additionally, fatigue strength is limited but their higher strength is significant in high cycle fatigue [11,12,13]. It is possible to use a wide range of materials such as stainless and tool steel, titanium alloys, aluminum, copper, nickel, or engineering ceramics [8,14].

The tested alloy is Ti-6Al-4V, which is a lightweight alloy with high strength properties. It is quite a good material for manufacturing thin wall structures [15,16]. The mechanical properties of the alloy depend on the phase arrangement as well as on the grain morphology. Shortly after the production process, as a result of the LENS process, the tested alloy is characterized by a fine-grain martensitic structure α′, as in the case of the SLM process [7]. The samples have an increased yield point and a tensile strength with reduced plasticity. In order to improve the LENS Ti-6Al-4V plasticity and anisotropic response, a heat treatment [7,10,13,17,18,19,20] is applied.

AM development contributed directly to the origin of a new group of functional materials, such as regular cellular structures. The mechanical properties of the structures shall be designed depending on their future application. A characteristic feature of the products is a low relative density and high strength [3,21,22,23,24]. Currently, there are many studies on energy-consuming structures produced using AM in various technologies, using a whole range of materials [2,21,23,25]. These structures are often inspired by nature, produced in the form of micro-trusses [21,26,27,28] or honeycomb structures [2,3,22,23,25], and their complex geometry would not be possible to obtain with conventional manufacturing methods. Energy-absorbing properties are closely related to the adopted topology of structures as well as processing parameters of the manufacturing process.

In the process of designing structures produced by additive techniques, numerical methods have been increasingly used to optimize the process parameters and the structure morphology in terms of expected utility features [3,8,23,24,25]. To develop an accurate numerical model, it is necessary to conduct a series of material tests to identify the basic material features. Through research of the microstructure, it is possible to take into account local effects (pores, inclusions, phase share) that have an impact on the global behavior of the whole structure [29,30,31,32,33,34,35].

The paper presents a comprehensive methodology for testing, modelling, and analyzing the Ti-6Al-4V alloy obtained by LENS technology, belonging to DED techniques. This technique is characterized by high density of a laser beam (thousands of J·mm^−1^) and a heat flow from a molten metal pool, which is controlled by conduction through a manufactured detail and the applied substrate, as well as by conduction through shielding gas and a nozzle supplying metal powder. It is possible to acquire a layer thickness of the level of 0.3–1 mm [11]. A proper selection of technological parameters and their impact on the obtained microstructure and mechanical properties [13,19,36,37] is also discussed. During the investigations, the relationship between the assumed wall thickness and the obtained microstructure [37,38], defect analysis, and fatigue strength of the samples [4,12,13,39] was analyzed.

The tests carried out using light microscopy aimed to determine the homogeneity of the microstructure of the analyzed alloy. Detailed structural studies were carried out on a scanning microscope equipped, among others, with an electron backscattered diffraction (EBSD), which allowed for a precise determination of the alloy phase composition, shape, and grain size. In addition, the fractures of the stretched samples were examined, which allowed linking of the obtained fracture type (brittle, plastic, or mixed) to the microstructure type (α′ or α + β) [12,13,39,40].

Measurements performed during the tensile test were supported by the Aramis system based on the digital image correlation (DIC) method. Such measurement equipment was successively used for measuring strains for specimens made of steel [41,42], wood [43], rubber [44], and foam [45]. It can be also adopted to observe the strains level within the tested structures, such as a seat belt, lattice structures, or composite joints [45,46]. This allowed for the determination of the plastic strain at the samples failure, which is impossible to be captured by means of traditional measurement methods (resistance tensometry and extensometry). The tensile test allows for the determination of the basic mechanical properties, such as Young’s modulus, yield strength, tensile strength, maximum elongation, and Poisson’s ratio. The obtained results were implemented into the constitutive material model to perform finite element analyses (FEA) of a uniaxial compression test of the selected energy-absorbing structures.

On the basis of the conducted tests, the obtained mechanical properties were confirmed and only slight differences were noted, due to the assumed technological parameters of the processes as well as the technology itself (SLM, EBM, LENS). These tests focused on identifying basic material features without indicating specific applications.

The paper is organized as follows: In Section 2 the specimens manufacturing process as well as the microstructural and tensile tests are described in detail. In Section 3, the results of uniaxial tensile tests and the microstructure photos are presented and discussed. The conclusions are included in the final section, Section 4.

## 2. Materials and Methods

### 2.1. Specimens Manufacturing Technology

**Figure 1:**
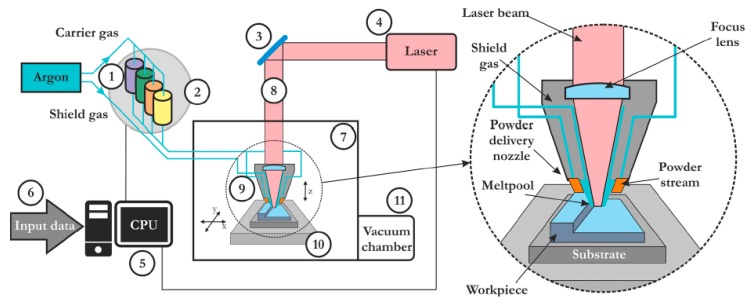
Laser engineered net shaping (LENS) system scheme [8].

**Figure 2:**
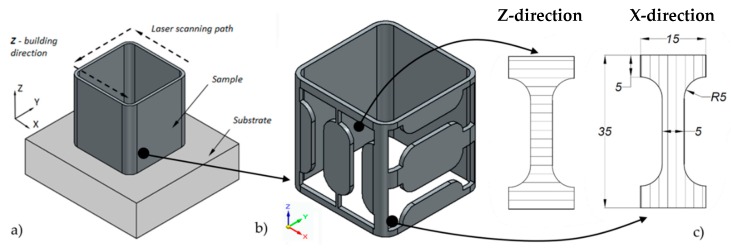
Strategy for manufacturing samples with the LENS technology: (**a**) Thin-walled square box; (**b**) method for cutting out specimens in two directions; and (**c**) geometry of the specimens with given dimensions.

The commercial Ti-6Al-4V powder, produced with an argon atomization method, was used for manufacturing the above mentioned square box structures. It was delivered by TLS Technik GmBH and Co (Bitterfeld-Wolfen, Germany) and the size of the particles was in the range of 44 to 105 μm. Optomec LENS MR 7 device (Albuquerque, NM, USA) with a 500 W laser (Figure 1) was used for the manufacturing of the thin-walled square boxes, with dimensions 44 mm × 44 mm × 40 mm (width × length × height) and walls’ thickness equal to 1.5 mm (Figure 2a). The Ti-6Al-4V substrate plate was sandblasted and degreased with acetone before manufacturing. The thin-walled components were manufactured with the process parameters determined experimentally and they are listed in Table 1.

One of the manufactured thin-walled boxes was subjected to heat treatment to obtain a two-phase structure and to improve the material ductility. The heat treatment process was conducted in a tubular furnace at low vacuum (×10^−2^ mbar). Before heating, the chamber was purged by flow of argon. The square box samples were annealed in a 1050 °C for 2 h and cooled down with the furnace. Heat treatment conditions were selected based on the previous studies [19,47]. The square box samples were annealed in a 1050 °C for 2 h and cooled down with the furnace.

After heating, the components were sandblasted to remove the surface impurities. The sandblasting process was performed using a sandblast machine P-05 (ZAP-BP, Kutno, Poland). The Al_2_O_3_ powder, with granulation of 90–150 μm, was applied as the abrasive agent and the surface of the details was sandblasted until it was matted.

The square-box geometry was developed in such a manner that the dumbbell specimens can be easily cut out in two directions: X-direction according to the laser beam scanning path and Z-direction consistent with the direction of the structure manufacturing (Figure 2a). The BP-97d electro discharge machining device (SEW MET, Ligota, Poland) was used for the cutting process. It is worth noticing that a smaller thickness was impossible to be obtained due to technological constraints. It was also required that the corner radius was large enough to not initiate cracking during tensile tests. Specimen geometry was designed to satisfy a 1:5 ratio of width to length measurement condition included in PN-EN ISO 6892-1. The specimen geometry was the smallest possible, in which the Aramis system could correctly measure the deformation. The dog bone samples were cut out in two directions to analyze the anisotropy of the material and its impact on the stiffness parameters of the samples [12,35,36]. The samples were tested for the material before and after heat treatment [16,34]. In addition, the roughness was measured, which allowed for the determination of the quality of the surface after production.

Before testing, each specimen was subjected to manual grinding with 120, 240, 600, and 1200 SiC papers, in order to eliminate crack initiator, due to a high roughness of the surface, which was measured using the Keyence VHX-6000 microscope (Keyence, Mechelen, Belgium) with a 1000 times magnification and with a special software (using depth from defocus algorithm, which enabled the obtainment of 3D information based on 2D image sharpness). Table 2 shows the results of measurements.

### 2.2. Microstructure Analysis

Before conducting the microstructural examination, the samples were subjected to grinding with 600–4000 SiC papers, polishing with 3 and 0.25 μm diamond suspensions, and final polishing with colloidal silica suspension. After performing the EBSD investigation, the samples were etched with the Kroll’s reagent to examine the microstructure with an optical microscope Nikon MA 200 (Nikon Metrology, CA, USA). To study the crystallographic orientation, imaging and texture were performed with EBSD FEI quanta 3D dual beam field emission gun scanning electron (FEI, OR, USA).

The fracture surface analyses were performed using secondary electron imaging on Quanta 3D FEI dual beam. Such a procedure allowed for qualitative evaluation, a fracture mode, and fracture surface details (e.g., defects, porosity).

### 2.3. Uniaxial Tensile Testing for Material Data Aquisition

**Figure 3:**
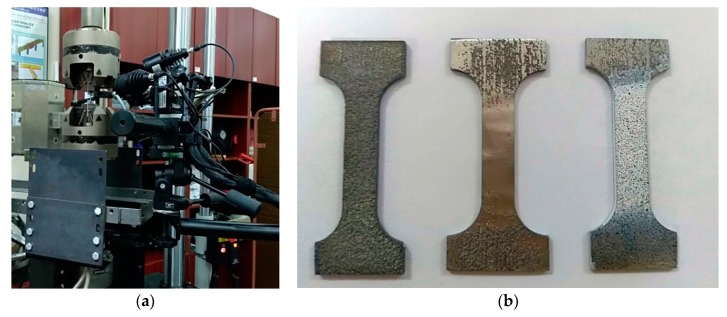
(**a**) Measuring position; and (**b**) preparation of the sample for test with digital image correlation (DIC) Aramis.

In order to identify the material parameters of the LENS Ti-6Al-4V alloy that had an impact on the behavior of energy absorbing structures under load conditions, a uniaxial tensile test was carried out according to PN-EN ISO 6892-1 standard, using a universal strength machine INSTRON 8862 at a room temperature of 23 °C, at a cross-head displacement rate of 1 mm/s. Twelve specimens (6 from each direction and 6 before and after heat treatment) were tested. Yield stress, ultimate tensile stress, elongation at failure, and Young’s modulus were determined using Aramis optical deformation measurement system and the measured cross-head displacement (Figure 3a). In Figure 3b, the surface condition of the specimen after cutting out from a thin square box, after mechanical grinding, and followed by stochastic pattern application on the measuring section (gauge length) is presented on the left, in the center, and on the right, respectively.

The advantages of using optical methods that provide a partial image of deformation distribution using DIC methods are associated with the ability to identify changes in the material structure at the micro-scale level. Based on the contrasting random surface texture of the side lock elements (pattern random point’s paint to the test object), the system divides the image into the working areas called facets. These facets can be correlated with the corresponding areas on the successive captured images. Subdivisions are of several pixels in size. The optical deformation measurement system compares the successive images to the photo taken before the initial application of the load. Subsequently, three-dimensional maps of displacements and deformations for all the facets were computed.

Owing to Aramis system, deformation of the tensile bars, the areas of minimum, and the maximum principal strain were determined. The Aramis optical-measurement system is designed for non-contact displacement measurements in both flat and spatial elements subjected to loading. It consists of a set of cameras registering the shape changes of the tested object and a suitably adapted and programmed computer that stores and processes the recorded images. Depending on the configuration (i.e., the number and speed of cameras), the system can be used to analyze the displacement and deformation fields of both flat or spatial elements subjected to static or dynamic load [42].

**Figure 4:**
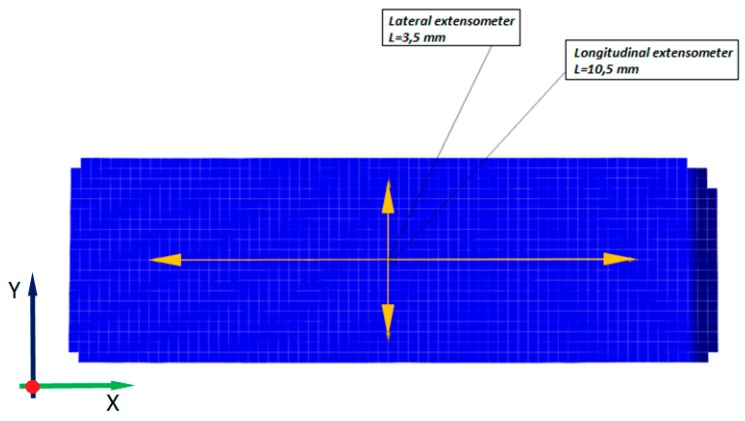
The specimen gauge length.

The optical system was calibrated for measurements using a calibration plate with dimensions of 30 × 24 mm before the test. This procedure enabled obtainment of the measurement area with dimensions of 35 × 26 mm, where the tested elements of the side lock were located. The lenses with a focal length of 50 mm were used. Cameras, distanced from the specimen front surface by 225 mm, registered the images with a frequency of 5 frames per second. In the analysis, the facets of 0.5 × 0.5 pixel size (about 0.05 × 0.05 mm) were employed. The surface of samples prepared to obtain a stochastic pattern is shown in Figure 3b. It was required to color the surface white, which is the background, and, in the next step, the specimen was sprayed with black dots, which created a random pattern. This allowed the system to identify each fragment of the specimen and to split the images into rectangular areas called facets, which were correlated with the corresponding areas on the other frames. The specimen gauge length was determined in the post-processor and amounted to approximately 10 mm (Figure 4). The results of strain measurements in Aramis system were synchronized in time with displacement and force signals from the testing machine (SATEC).

Logarithmic strains are computed locally in X- and Y-directions of the selected coordinate system in each photo as follows [42,46]:

(1) $$\varepsilon_{log}=\log\lambda$$

The value of *λ* is calculated based on the following formula:

(2) $$\lambda =\underset{l\to 0}{\lim}\frac{l+\Delta l}{l}$$

Symbol *l*_0_ denotes the initial distance between two neighboring facets and Δ*l* is the distance increased during the test.

Engineering strains are computed locally in X- and Y-directions of the selected coordinate system in each photo as follows:

(3) $$\varepsilon_{eng}=\lambda -1$$

A coordinate system, in which X-axis extends along the load axis of the sample, was assumed. The measurement field was selected in the middle part of the sample. 

The local value of the longitudinal and transverse strains was averaged within the area of measurement and used for further calculations, as well as for drawing the graphs of the tensile curves. Actual engineering stress values were calculated according to the following equation:

(4) $$\sigma_{eng}=\frac{P}{A_{0}}$$

where *P*—current value of the tensile force, *A*_0_—initial cross-section.

Tensile strength was determined according to the following equation:

(5) $$R_{m}=\frac{P_{max}}{A_{0}}$$

where *P_max_* is the maximum force registered during the test.

## 3. Results and Discussion

### 3.1. Microstructure Analysis of As-Built and after Heat-Treatment LENS Ti-6Al-4V

**Figure 5:**
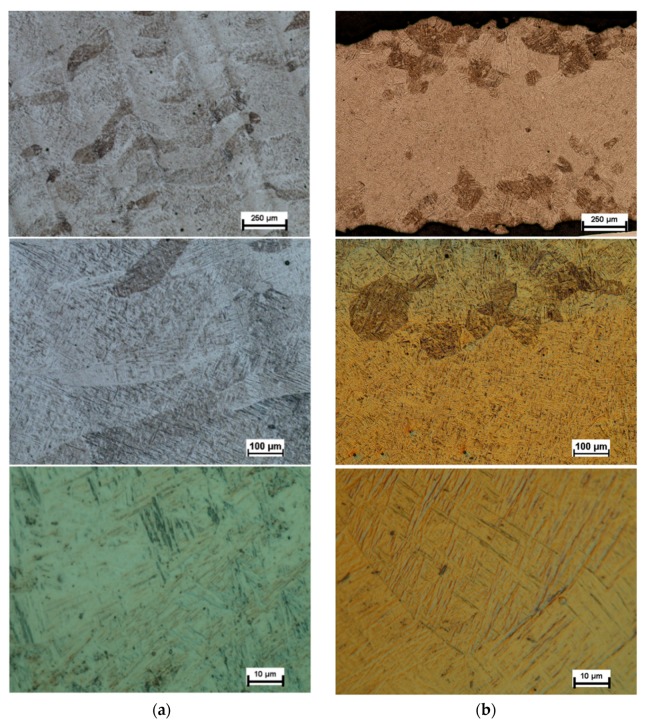
Optical micrographs of Ti-6Al-4V microstructure as-built: (**a**) longitudinal section; and (**b**) cross-section.

In the LENS process, application of the successive powder layers re-melts the grains, which serve as seeds, causing the epitaxial growth of subsequent columnar grains. Figure 5 shows the results of optical microscopy exam microstructure of the alloy before heat treatment in the longitudinal section (Figure 5a) and in the cross-section (Figure 5b). The analyzed samples were characterized by a coniferous microstructure, which was formed, first of all, on the grain boundaries of β phase and gradually filled up the entire grain space. The obtained microstructure of LENS Ti-6Al-4V is similar to a microstructure reported during research [10,11,13].

This microstructure is a characteristic feature of α′ phase, which is defined in the literature as a consequence of α phase hardening [9]. Obtainment of the microstructure is possible during fast cooling above the critical rate which for this type of material is at the level of about 1000 K/s [48]. The cooling rate above 1000 K/s is characteristic for processes using laser radiation, including LENS methods. Then, β phase is transformed directly into α′ phase, instead of α + β phase, as it is the case of the furnace cooling.

The analysis of the microstructure at low magnification allowed for the observation of the original β phase boundaries. The prior β grains had long axes of 0.25 to 0.75 mm, aligned with the build direction, and short axes from 0.2 to 0.3 mm in width. The conducted comprehensive literature study on the primary β-grain structure and texture demonstrated a strong 〈001〉β//Nz fiber texture of prior β grains, with the longitudinal axis parallel to the building direction in Ti-6Al-4V alloy fabricated by additive manufacturing [11,49,50,51]. Moreover, the α′ phase of the as-built components had a weak texture because of the relatively high number of variants that precipitate within each columnar β grain [50].

**Figure 6:**
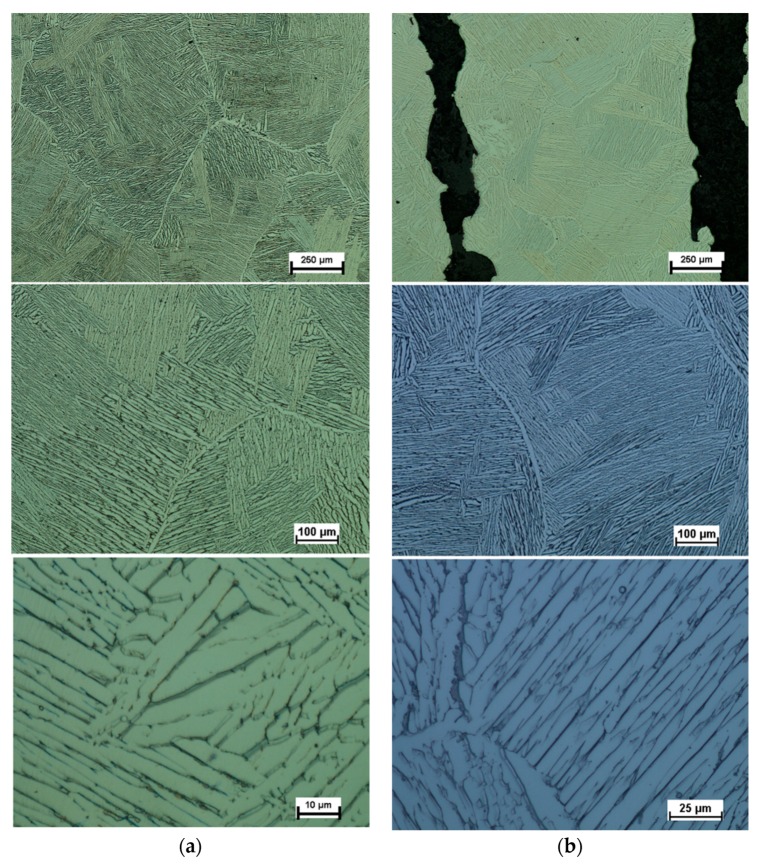
Optical micrographs of Ti-6Al-4V microstructure after heat-treatment: (**a**) longitudinal section; and (**b**) cross-section.

Figure 6 shows the microstructure of the alloy after heat treatment examined by optical microscopy. In this case, the presence of a two-phase structure is clearly visible and results are corresponding with those reported in [10,52]. The microstructure contains α-phase lamellas (white areas) and β-phase lamellas (black areas). Visible primary grains of the β-phase indicate a clear growth of these grains during the applied heat treatment. The barely visible black spots are few small pores.

Strongly-textured structures can lead to significant anisotropic mechanical properties causing different mechanical responses to the external loading along different sample orientations [50].

**Figure 7:**
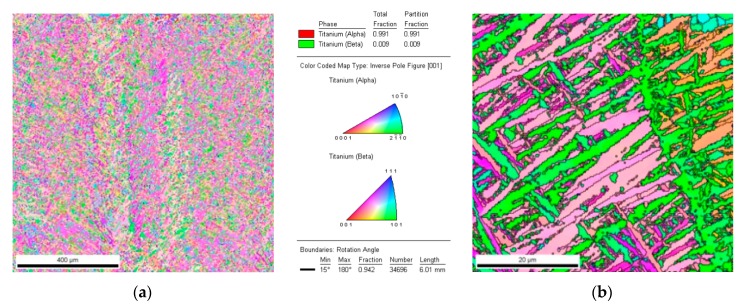
Electron Backscatter Diffraction (EBSD) orientation map of LENS Ti-6Al-4V (**a**) as-built; and (**b**) grain boundary reconstruction.

The EBSD data produced the inverse pole figure maps, pole figures, and orientation distribution functions (represented by generalized spherical harmonics). Figure 7 illustrates an EBSD orientation map of the LENS Ti-6Al-4V as-built. EBSD analysis proved the absence of β phase (phase fraction below 1%) in the Ti-6Al-4V alloy structure immediately after production. The martensite needles with different crystallographic orientations are clearly visible. The presence of wide-angle boundaries (above 15°) was detected. It means that a single grain was martensite needle, whose typical structure and morphology are shown in Figure 7b.

**Figure 8:**
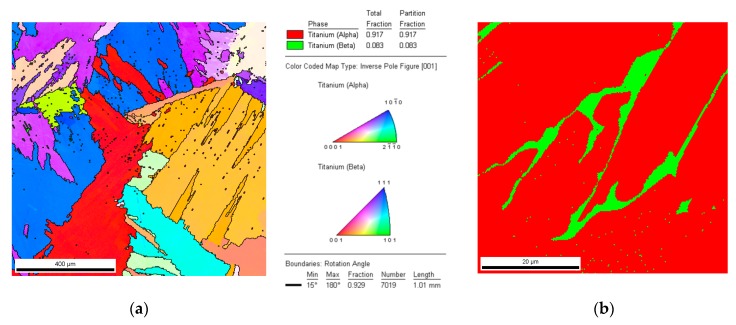
EBSD orientation map of LENS Ti-6Al-4V (**a**) after heat treatment; and (**b**) phase distribution map.

Figure 8 presents the EBSD orientation map of the LENS Ti-6Al-4V after heat treatment. The data obtained from EBSD analysis proved the presence of both α and β phase in the samples after heat treatment. The map of the phase distribution presented in Figure 8b shows that the amount of β phase was clearly smaller than α phase, which was about 8%.

### 3.2. Uniaxial Tensile Testing of As-Built and after Heat-Treatment LENS Ti-6Al-4V Material

**Figure 9:**
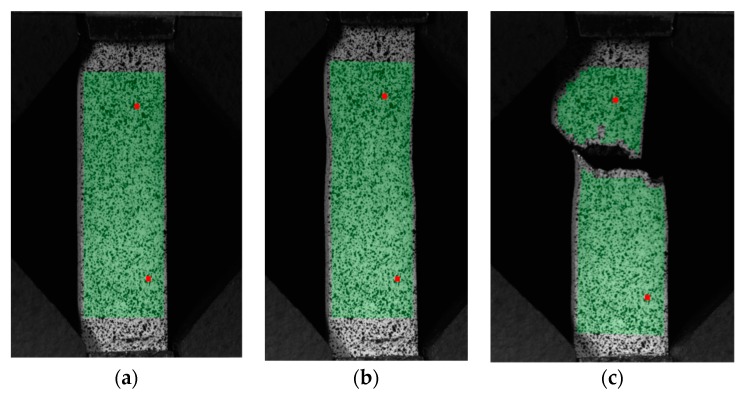
Picture of the specimen as-built with the analyzed area, marked with a green color: (**a**) Stage “0” at the beginning of the test; (**b**) stage in the middle of the test; and (**c**) stage registered in the first frame after failure.

Based on Aramis measurements, local axial strains and local shear strains, which occurred in the plasticized area of the neck, were obtained. Figure 9 presents the selected stages of the tensile test. Figure 9a presents an unloaded specimen at the beginning of the test, Figure 9b presents the stage before the failure and necking is also observed. The final stage is illustrated in Figure 9c and presents specimens after failure. Within the analyzed area, marked with a green color, local strain in facets were measured.

**Figure 10:**
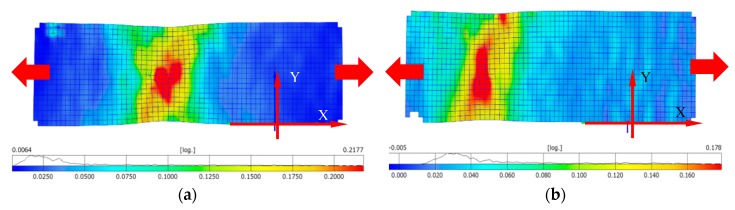
Map of principal strain before failure in specimen as-built: (**a**) X-direction; and (**b**) Z-direction.

Distribution of principal logarithmic strains are presented in Figure 10a,b, which correspond to the photos taken on frame before failure (Figure 9b). Local axes are also included in the results. Figure 10a presents specimen as-built stretched at X-direction and Figure 10b presents the same results for the material manufactured in Z-direction. Plastic strain for X-direction was approximately 21.7% before failure and for Z-direction about 17%. The Z-direction specimen failed near the grip.

**Figure 11:**
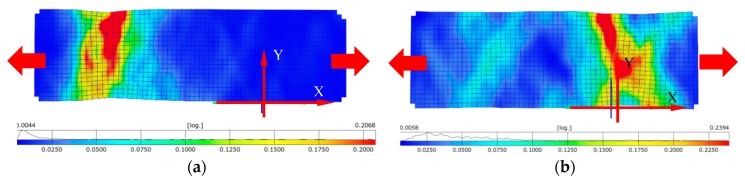
Map of principal strain before failure in the specimen after heat treatment: (**a**) X-direction; and (**b**) Z-direction.

Figure 11a presents the specimen after heat treatment stretched in the X-direction and Figure 11b presents stretching it in the Z-direction. Plastic strain for the X-direction was approximately 20.6% before failure and for the Z-direction it was about 24%. The strain at fracture for the Z-direction increased compared to the sample before heat treatment. Strain concentration the specimens after heat treatment occurred outside their central part, compared to as-built specimens with central strain concentration.

**Figure 12:**
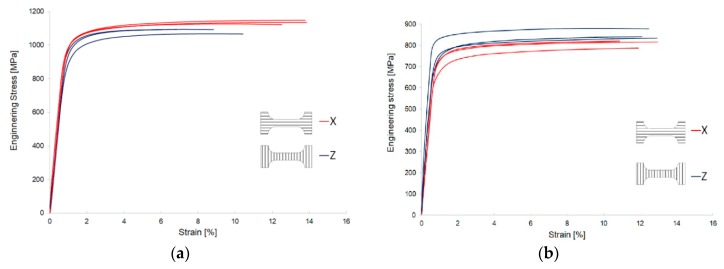
Engineering tensile stress–strain curves of the LENS Ti-6Al-4V: (**a**) As-built; and (**b**) after heat treatment.

In Figure 12, engineering stress vs. strain characteristics, calculated from the uniaxial tests, are presented for X and Z specimens. The results before and after heat treatment are shown in Figure 12a,b, respectively. For the as-built specimens, X-direction of cutting resulted in slightly higher ductility, whereas in the Z-direction an opposite correlation was observed. A visible influence of the heat process was observed in the decreasing of strength while increasing the ductility.

The results obtained from the tensile test of LENS Ti-6Al-4V as-built are presented in Table 3. The sample direction (X vs. Z) significantly influenced the mechanical properties. The elongation of the Z-direction sample was on average 30% lower compared to the X-direction sample. Stiffness of the X-direction sample was about 8% higher than in the case of the Z-direction sample. The value of effective plastic strain was read from Figure 10a,b.

The results obtained from the tensile test of LENS Ti-6Al-4V after heat treatment are presented in Table 4. The evaluated heat treatment clearly influences all the measured properties and; therefore, it can be concluded that the anisotropic features were minimized. After heat treatment, a significant increase in ductility was observed as a result of α′ decomposition into α + β structure. The value of effective plastic strain was read from Figure 11a,b.

Table 5 compares the results of tests available in literature. There were selected positions, in which the specimens of similar geometry and dimensions were uniaxially stretched. Additionally, the loading direction was defined and denoted according to terminology adopted in the article. The data included in the Table 5 show that there was anisotropy of mechanical properties in the specimens and the greatest differences were observed on their elongation. There was also observed an influence of heat treatment owing to which an anisotropy effect in the structure was reduced and larger elongation of the specimens was achieved.

On the bases of summarization in Table 5, it can be concluded that the obtained results are comparable with data reported in literature.

### 3.3. Fracture Mechanism

**Figure 13:**
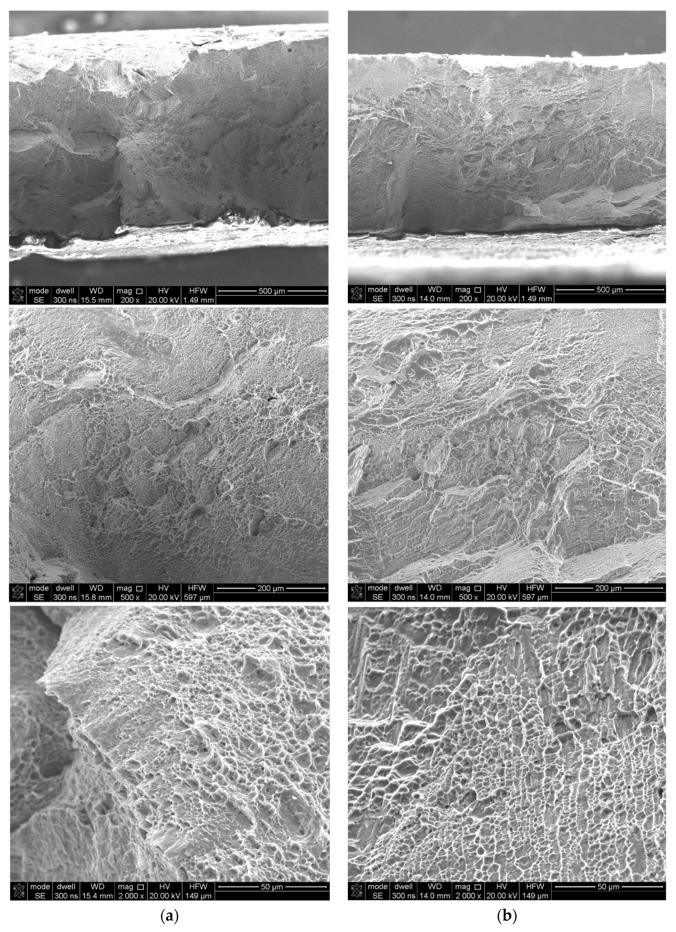
Fracture surfaces of the LENS Ti-6Al-4V longitudinal section: (**a**) As-built; and (**b**) after heat treatment.

**Figure 14:**
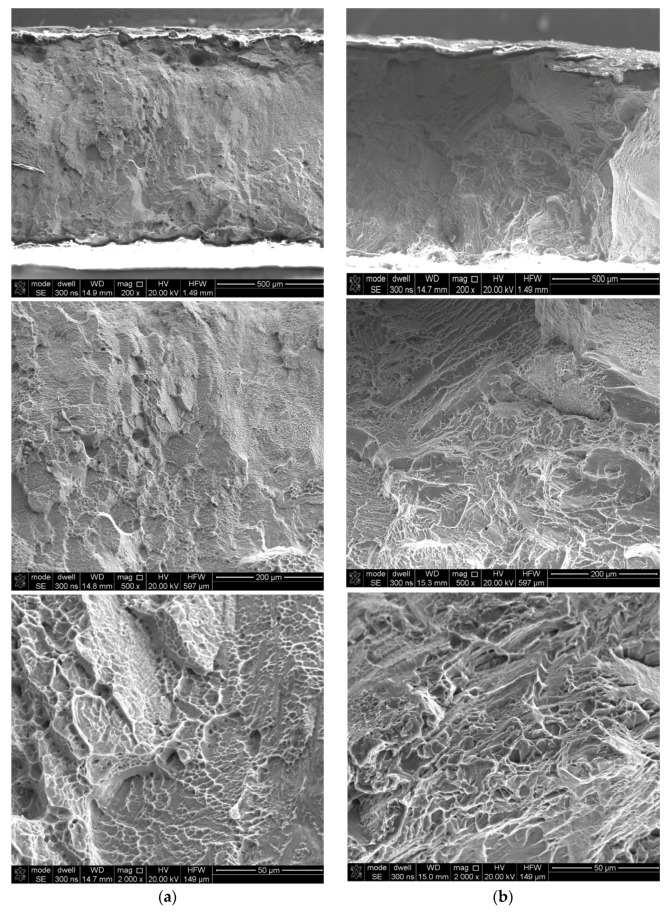
Fracture surfaces of the LENS Ti-6Al-4V cross section (**a**) as-built (**b**) after heat treatment.

Fracture surface analyses were conducted using secondary electron imaging on a scanning microscope. In the case of as-built samples, a brittle-ductile fracture, with a predominantly brittle fracture, was obtained (Figure 13a). Although the dimple rupture dominated, quasi-cleavage facets features of a brittle type were observed. After the tensile tests, the fracture surfaces in the as-built and stress-relieved samples resembled a “cup and cone”, which is a characteristic for ductile types of the fractures (Figure 13b and Figure 14b). The presented fractographs are similar to the previous studies [10,11].

### 3.4. Constitutive Modelling with FEA

**Figure 15:**
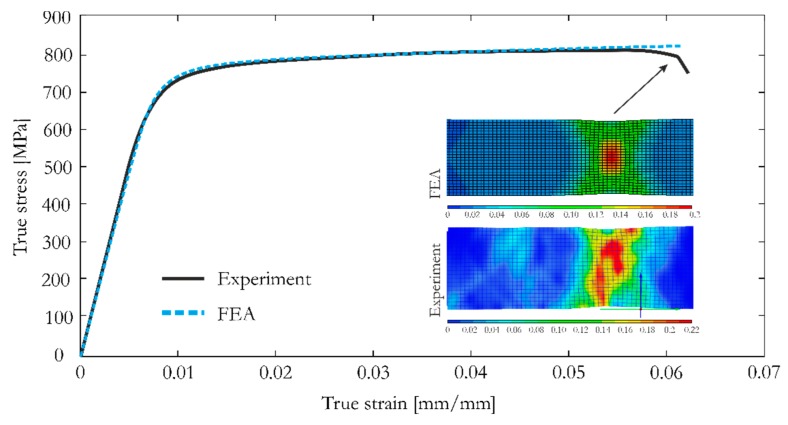
Correlation of the constitutive model based on true stress vs. true strain curves and strain distribution within the specimen neck: Finite Element Analysis (FEA) and experiment.

The discussed material data was used for determining the parameters for the selected constitutive model. An elasto-visco-plastic material model was adopted for predicting the Ti-6Al-4V behavior. In the first step of FEA, the material model was correlated based on the uniaxial tensile tests. The elasticity modulus, Poisson’s ratio, yield stress, and EPFS were determined from an experimental uniaxial tensile test as the average values from the two directions of sample (see Table 4). The adopted constitutive model uses an effective stress (ES) vs. effective plastic strain (EPS) curve, in which the last point corresponds to experimental values of ultimate stress and EPFS. The results obtained from the numerical simulation of the uniaxial tensile tests were compared with the Aramis measurements (Figure 15). The elastic and plastic ranges were in excellent agreement with the experimental curve. Moreover, strain distributions within the neck of the dumbbell specimen obtained from FEA and Aramis measurement were also very similar. This indicates that the constitutive model was correct and could be applied for simulating the cellular structure.

Obtainment of the correlated material model was necessary to carry out numerical simulation of the compression tests for a selected honeycomb cellular structure, with a geometry similar to a cube with dimensions as follows: width: 40 mm, height: 40 mm, and thickness: 10 mm. The wall thickness was approximately 0.7 mm and the elementary cell diameter was 5 mm. The results of the FEA were compared with the outcomes from the actual tests, which were conducted on a universal MTS Criterion C45 strength machine (MTS, MN, USA). During the experimental test the loading velocity was 1 mm/s. The honeycomb structure was compressed until reaching 50% of its height. For recording the structure deformation during experimental testing, the fast Phantom V12 camera (Vision Research, NJ, USA) was used.

**Figure 16:**
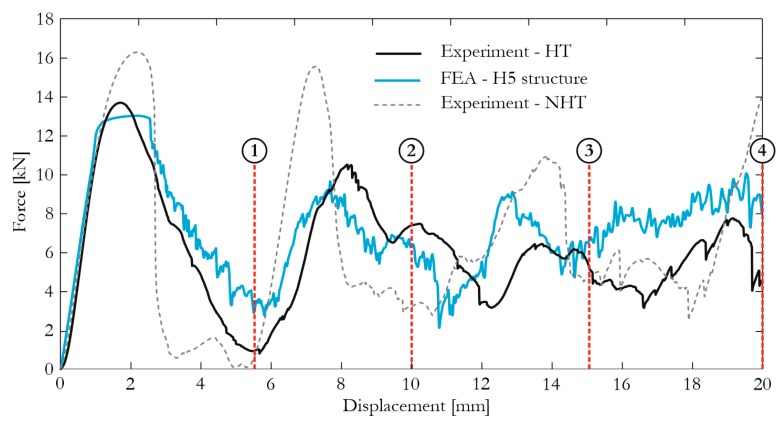
Force characteristics obtained from FEA and experiments for the H5 structure [25] (Elsevier, 2018, Additive Manufacturing).

**Figure 17:**
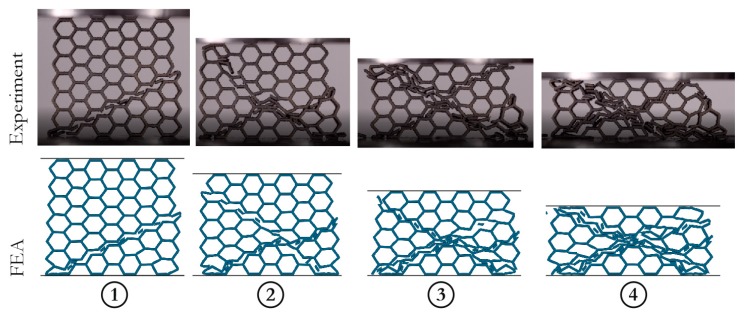
Selected time frames of the H5 structure compression process: Comparison between the FEA and the experiment [25] (Elsevier, 2018, Additive Manufacturing).

The results of experimental tests and numerical simulations were compared. In Figure 16, force characteristics are presented, and the experiments with the honeycomb structure after (depicted as HT) and without the heat treatment (depicted as NHT) are also included. A satisfactory reproduction of the actual results can be observed, and the influence of heat-treatment is visible, resulting in a more brittle fracture of the structure with no heat-treatment effect. Thus, the obtained results confirmed the observations during the tensile test (see Section 3.2). In Figure 17, the comparison between the deformations of the structure observed during experimental and numerical tests are presented. Four selected time frames of the compression process were considered for comparison purposes. An excellent reproduction of the structure behavior can be observed with a similar fracture characteristic in all stages. The FEA results proved that the discrete representation and the constitutive model, with the properties obtained from the discussed uniaxial tensile tests, were correctly developed.

A detailed description of the FE model, including adopted modeling techniques, methodology for determining properties for the constitutive model, and a validation procedure can be found in [25].

## 4. Conclusions

In this study, specimens for tensile test, cut in two build orientations, were evaluated in two heat treatment conditions: the as-built condition and after heat treatment. Their microstructure as well as mechanical properties were evaluated. For this purpose, a tensile test was carried out, which showed that both the direction of the load and the heat treatment conditions affect the mechanical properties of the alloy.

- In the case of a microstructure, there was a clear difference between as-built and after heat treatment specimens. In both conditions, prior-β grains were found to be aligned with the manufacturing direction. Ti-6Al-4V fabricated by LENS consists of columnar prior-β grains filled with acicular α martensite, and showed high yield strength with reduced plasticity compared with alloy manufactured conventionally.
- The analysis of the results obtained from the static tensile test indicated a significant influence of the as-built sample loading direction, since specimen elongation in the Z-direction was 30% smaller.
- The results show a significant influence of heat treatment, both on the alloy microstructure and its mechanical properties. Obtainment of a dual-phase structure increased specimen plasticity and reduced anisotropy of mechanical properties along, with a decrease of yield stress and ultimate tensile stress.
- It was noticed that in the Ti-6Al-4V alloy obtained by traditional methods, ductile fractures were obtained in which the characteristic elements of the brittle fracture are not visible. This difference is due to the existence of α′ martensite, which is characterized by higher durability but worse plasticity. The partial re-melting of the previous layer of applied powder caused the grain to lengthen according to the direction of the model’s production.
- The adopted numerical methodology proved to be suitable for simulating energy-absorbing cellular structures. Furthermore, the implemented constitutive model described, based on the experimental material tests, resulted in a reasonable reproduction of the material behavior during the deformation process. Application of numerical methods enabled the reduction of time consuming and expensive experimental methods.

In future studies, obtained results will be used to create a large-scale cell structure model from the LENS Ti-6Al-4V alloy subjected to a compressive load. Additionally, knowing the technological limitations, the geometry of the structure will be optimized in terms of maximizing its energy consumption while maintaining the minimum weight.

## Figures and Tables

**Table 1:** Process parameters.

Process Parameters	Value
Laser power (W)	400
Powder flow rate (rpm)	11.5
Layer thickness (μm)	30
Feed rate (mm/s)	20
Oxygen concentration (ppm)	>5

**Table 2:** Surface roughness of the LENS samples.

Surface Roughness Ra (µm)	Value
As-built X-direction	50 ± 10
As-built Z-direction	48 ± 8
After heat-treatment X-direction	40 ± 5
After heat-treatment Z-direction	43 ± 3
After polishing	8 ± 4

**Table 3:** Tensile properties of LENS Ti-6Al-4V as-built.

Direction	Yield Stress σ_y_ (MPa)	Ultimate Tensile Stress UTS (MPa)	Elongation ε (%)	Effective Plastic Strain EPFS (%)	Young’s Modulus E (GPa)
X	1080 ± 4	1139 ± 12	13.1 ± 0.7	21.7	118 ± 2
Z	1038 ± 28	1080 ±13	8.7 ± 1.8	17.8	109 ± 5

**Table 4:** Tensile properties of LENS Ti-6Al-4V after heat treatment.

Direction	Yield Stress σ_y_ (MPa)	Ultimate Tensile Stress UTS (MPa)	Elongation ε (%)	Effective Plastic Strain EPFS (%)	Young’s Modulus E (GPa)
X	762 ± 20	813 ± 16	13.8 ± 2.3	20.6	100 ± 4
Z	808 ± 38	858 ± 27	14.3 ± 3.5	23.9	108 ± 10

**Table 5:** Tensile properties of Ti-6Al-4V obtained with different AM techniques.

Method		Geometry of Sample	Direction	Yield Stress σ_y_ (MPa)	Ultimate Tensile Stress UTS (MPa)	Elongation ε (%)
Direct Energy Deposition (DED) [11]	As-built	Tall wall	X	960 ± 26	1063 ± 20	13.3 ± 1.8
			Z	945 ± 13	1041 ± 12	18.7 ± 1.7
Powder Bed Fusion—Selective Laser Melting (SLM) [50]	As-built	Dog bone	X	978 ± 5	1143 ± 6	11.8 ± 0.5
	Heat treated		Z	967 ± 10	1117 ± 49	8.9 ± 0.4
			X	958 ± 6	1057 ± 8	12.4 ± 0.7
			Z	937 ± 9	1052 ± 11	9.6 ± 0.9
Electron Beam Melting (EBM) [10]	As-built	Dog bone	X	1006	1066	15
			Z	1051	1116	11
	Heat treated		-	1039	1294	10
